# Oral Administration of Apigenin Inhibits Metastasis through AKT/P70S6K1/MMP-9 Pathway in Orthotopic Ovarian Tumor Model

**DOI:** 10.3390/ijms13067271

**Published:** 2012-06-13

**Authors:** Jun He, Qing Xu, Min Wang, Chongyong Li, Xu Qian, Zhumei Shi, Ling-Zhi Liu, Bing-Hua Jiang

**Affiliations:** 1 State Key Lab of Reproductive Medicine, and Department of Pathology, Cancer Center, Nanjing Medical University, Nanjing, 210029, China; E-Mails: jun.he@jefferson.edu (J.H.); qing.xu@jefferson.edu (Q.X.); wangmin1351@126.com (M.W.); lichy100@yahoo.com.cn (C.L.); taurusgoldox@163.com (X.Q.); shizhumei2008@sohu.com (Z.S.); 2 Department of Pathology, Anatomy and Cell Biology, Thomas Jefferson University, Philadelphia, PA 19107, USA; E-Mail: ling-zhi.liu@jefferson.edu

**Keywords:** apigenin, orthotopic ovarian cancer model, metastasis, MMP-9

## Abstract

Apigenin, a flavonoid commonly present in the daily diet, is known for its potential anti-tumor properties. However, the effect of apigenin via oral administration on tumor growth and metastasis remains unknown. In this study we developed an orthotopic ovarian tumor model in nude mice to test the effect of apigenin oral administration, and showed that apigenin inhibited the micrometastasis of cancer cells in the animal tumor model. To understand the mechanism of apigenin in inhibiting metastasis, we found that apigenin greatly inhibited MMP-9 expression and p-AKT and p-p70S6K1 levels in the tumor tissues compared to the control group. We further demonstrated that the downregulation of MMP-9 by apigenin was mediated by the AKT/p70S6K1 pathway. These findings help to address the question with common interests to the public of whether oral uptake of flavonoids is effective in preventing cancer. Our results demonstrate for the first time that oral uptake of apigenin can inhibit tumor metastasis through MMP-9 expression using the orthotopic ovarian tumor model.

## 1. Introduction

Ovarian cancer is a common female tumor worldwide, and has the highest mortality rate among all gynecological malignancies [1]. Due to difficulties in early diagnosis, chemotherapy is the only treatment of choice for the majority of ovarian cancer patients. However, clinical experience suggests that patients are prone to developing chemoresistance toward the commonly used anti-cancer agents, such as cisplatin, thus directly diminishing successful chemotherapy. Therefore, the search for new alternative agents with a preventive or therapeutic effect is important for ovarian cancer treatment. Apigenin (4′,5,7-trihydroxyflavone) is a dietary flavonoid commonly found in many fruits and vegetables [2]. We previously found that apigenin inhibits tumor growth and angiogenesis induced by different cancer cells [3–5]. Several other laboratories have also demonstrated that apigenin has antiproliferative and anti-tumor properties in pancreas, prostate and colon cancer cells [6–8]. However, it is unknown whether the oral uptake of apigenin has any effect in inhibiting tumor growth and metastasis due to the general concern of flavonoid bioavailability.

Activation of PI3K/AKT and Ras/MAPK is associated with increased cell scattering, cell motility, proliferation and survival [9]. Our previous studies have indicated that the PI3K/AKT/mTOR/p70S6K1 pathway is critical for ovarian cancer cell proliferation, migration and invasion [10]. We also demonstrate that apigenin inhibits cancer cell proliferation by blocking the PI3K/AKT pathway in ovarian cancer and lung cancer cells [3,11]. Other studies have shown that apigenin can impair cancer cell motility by suppressing focal adhesion kinase/Src signaling, a key step in the development of tumors and ultimately metastasis [12]. Since many fruits and vegetables contain apigenin, there is general public interest as to whether oral uptake of apigenin has any anti-cancer effect. To answer this question, we developed an orthotopic ovarian cancer model by implanting OVCAR-3 cells into the ovaries of nude mice to determine whether apigenin oral administration has any effect on tumor growth and metastasis.

## 2. Results and Discussion

### 2.1. Establishment of the Orthotopic Ovarian Tumor Model in Nude Mice

**Figure 1:**
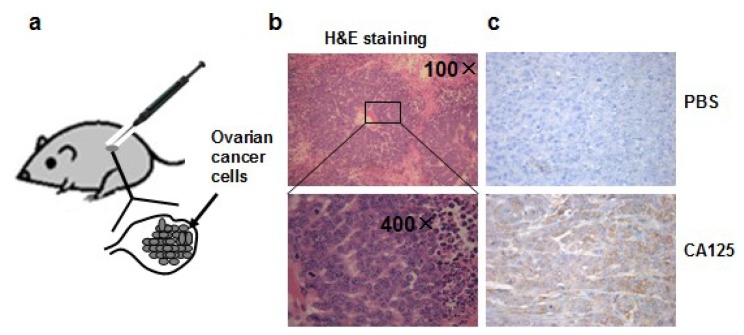
(**a**) Orthotopic ovarian tumor model in this study; (**b**) H&E staining of tumor section showing tumor cells and surrounding tissue; (**c**) Expression of ovarian cancer biomarker CA125 detected by immunohistochemical staining. Upper panel: section was incubated with PBS buffer instead of CA125 primary antibody for immunohistochemical staining (200× magnifications), bottom panel: section incubated with CA125 antibody.

To mimic normal ovarian cancer development, we injected ovarian epithelial cancer cells OVCAR-3 cells, which are featured by strong tumorigenicity and resistance to chemotherapy, into the ovaries of nude mice, to develop an orthotopic ovarian tumor model. In this orthotopic ovarian tumor model, we grow cancer cells in the natural ovary environment relevant to normal tumor development in which bioactivity and pharmacological kinetics are similar to those of the human ovarian tumor development (Figure 1a). All the mice had palpable tumors in the abdominal cavity four weeks after the implantation of OVCAR-3 cells into ovaries. The tumors were collected, fixed in Bouin’s solution, embedded in paraffin, and stained with hematoxylin and eosin, which showed the characteristic structure of ovarian cystadenocarcinoma (Figure 1b). The levels of CA125 expression, a biomarker of ovarian cancer, were greatly elevated in the tumor tissues using immunohistochemistry analysis (Figure 1c), confirming the successful generation of the ovarian tumor model.

### 2.2. Apigenin Did not Significantly Inhibit Tumor Growth, but Inhibited the Occurrence of Micrometastasis in the Small Intestine and Stomach

**Figure 2:**
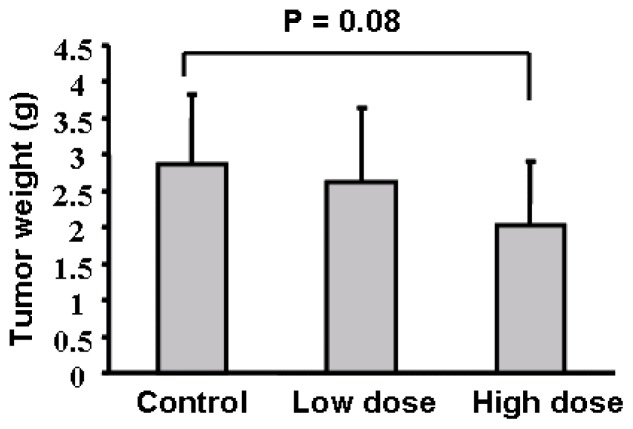
Tumor growth assay. Average tumor weights per mouse for each group were presented as mean ± SD when the mice were euthanized in 4 weeks (*n* = 10).

To test the effect of apigenin oral administration on tumor development, we divided 30 nude mice into three groups, gave the mice with 0.2% gelatin solution, 75 mg/kg and 150 mg/kg apigenin dissolved in 0.2% gelatin solution, respectively, by intragastric administration daily for one week before injecting ovarian cancer cells into the ovaries of mice. Apigenin at 75 mg/kg and 150 mg/kg respectively represent 0.025% and 0.05% of food uptake daily by the mice. Oral administration of the solvent and apigenin was continued as above until the mice were sacrificed. The mouse body weight was measured every week. No significant difference was found regarding the averages of mouse body weight among the three different groups (data not shown). We found that apigenin treatment appeared to inhibit tumor growth; but the differences among the control, low dose, and high dose treatments were not statistically significant (Figure 2). Micrometastasis is an early event in cancer dissemination. To examine the micrometastases of human ovarian cancer cells in the host mice, we performed a metastasis assay based on genomic PCR analysis for Alu sequences. Alu insertion elements are the most numerous short interspersed repeats in the human genome discovered in the mid-1980s [13]. Alu sequences are specific to the human genome and this can be utilized to evaluate the metastatic potential of human cancer cells in different tissues and organs without the interference of mouse cells. To analyze the sensitivity of the PCR-based assay, we found that a total of 100 OVCAR-3 cells in one million murine cells were detectable by the assay (data not shown). We analyzed the Alu sequences in liver, lung, small intestine and stomach of the nude mice. The results in Table 1 showed whether human ovarian cancer cells can be detected in the 10 mice for each group in four different organs. For example, ovarian cancer cells were detected in the small intestine in seven mice in the solvent control group, and were also detected in five and three mice respectively in the low and high dose apigenin-treated groups. There are significant differences of micrometastasis between the control and high dose apigenin-treated group. Similarly the differences of micrometastasis between the control and the high dose apigenin-treated group are statistically significant in the stomach tissues (Table 1).

### 2.3. Apigenin Significantly Decreased the Number of Metastasis Cancer Cells Detected in Several Organs

**Figure 3:**
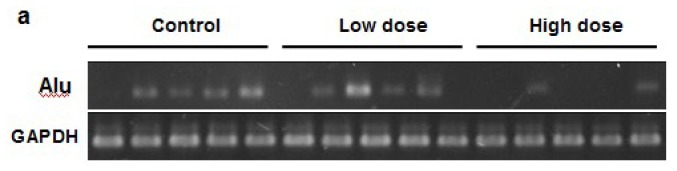

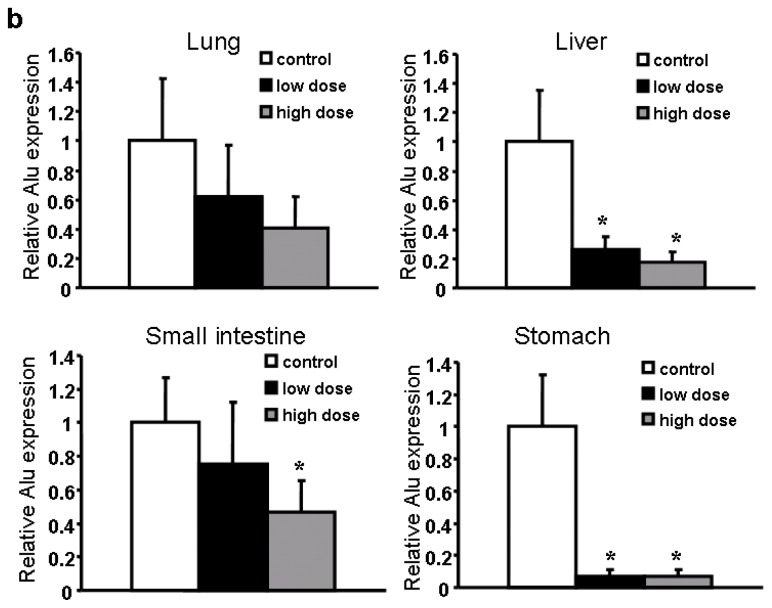
Detection of Cancer Cells in Different Organs. (**a**) Representative Alu bands in liver tissues; (**b**) Quantification of Alu expression in four organs from three groups (*n* = 10).

To determine the amount of micrometastasis in the organs, we quantified the Alu expression levels in different tissue samples. Low doses of apigenin treatment significantly decrease the magnitude of micrometastasis in the liver and stomach, while high doses of apigenin treatment greatly inhibited the micrometastasis in all the four organs tested (Figure 3a,b). Collectively, our *in vivo* experiments indicate that apigenin inhibits ovarian cancer micrometastasis. As apigenin was administered via the oral route, it indicates that oral uptake of apigenin also has an effect in inhibiting tumor metastasis. Thus, it would be interesting to further investigate the metabolism and pharmacokinetics of apigenin after oral administration in the future.

### 2.4. Apigenin Inhibited OVCAR-3 Cell Migration and Invasion

**Figure 4:**
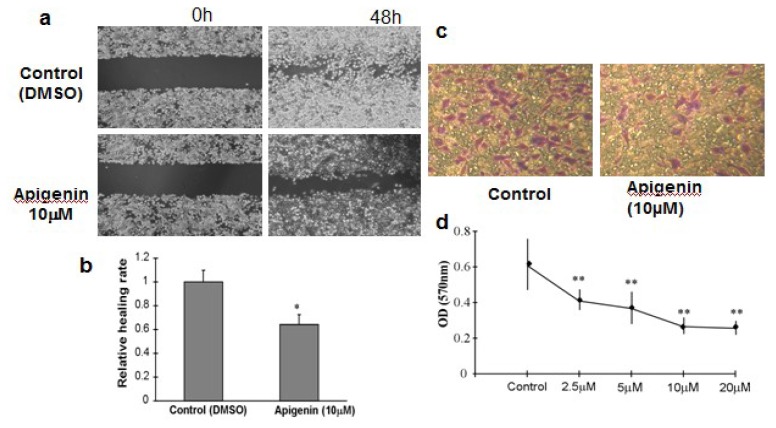
Cell Migration and Invasion Assay. (**a**) OVCAR-3 cells were cultured to 90% confluence. A sterile 200 μL pipette tip was used to scratch the cells to form a wound. The cells were then washed with PBS buffer, and cultured in RPMI 1640 medium containing 10% FBS with 10 μM apigenin for 48 h. The cells treated with solvent alone were used as control. The migration of the cells to the wound was visualized with an inverted Olympus phase-contrast microscope. The representative fields were photographed; (**b**) The relative healing rates were quantified with measurements of the gap sizes after the culture, and normalized to that of control. Three different areas in each assay were chosen to measure the distances of migrating cells to the origin of the wound. The mean values and the standard deviation were obtained from three experiments; (**c**) The representative images of invasive cells at the bottom of the membrane stained with crystal violet were visualized as shown; (**d**) The stained cells were eluted by 20% glacial acetic acid for 20 min. The elution was measured at 570 nm to obtain the OD570 values, and normalized to that of control. Each treatment had five replicates. All tests were performed in triplicate and presented as mean ± SD. * indicates significant difference (*p* < 0.05, One-Way ANOVA).

The metastatic process involves cell scattering, motility, ECM degradation, migration and invasion through the basement membranes [14]. To examine whether apigenin affects ovarian cancer cell migration *in vitro*, we performed wound-healing experiments using OVCAR-3 cells and measured the distance of cell migration to the wound area. We observed a significantly slower wound-healing rate in the cells treated with 10 μM apigenin for 48 h compared to the control cells (Figure 4a,b). There was no significant difference in cell viability between control cells and apigenin-treated cells (data not shown). The invasiveness of tumor cells represents the potential metastatic capacity. We used the Transwell assay to evaluate the invasiveness by counting the number of cell invasions through the Matrigel layer. Apigenin treatment inhibited OVCAR-3 invasion in a dose-dependent manner (Figure 4c,d).

### 2.5. Apigenin Inhibited MMP-9 Expression through AKT/p70S6K1 Pathway

**Figure 5:**
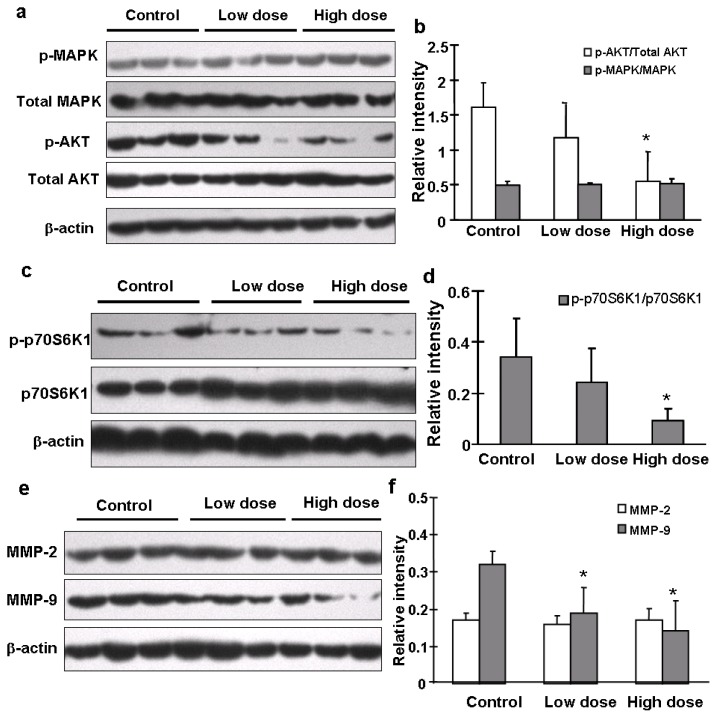

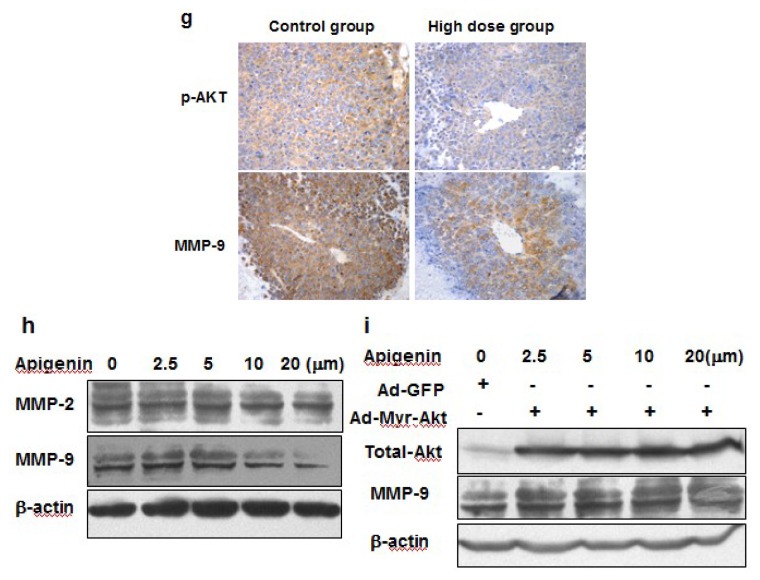
Signaling molecules affected by apigenin treatment. (**a**–**f**) Levels of p44/42-MAPK, total MAPK, p-AKT, total AKT, p-p70S6K1, total p70S6K1, MMP-2 and MMP-9 proteins in tumor tissues were analyzed by immunoblotting as described in Materials and Methods. The representative bands from three samples in each group are shown. Scanning densitometry was used for quantification of specific protein signals. Relative expression levels of p-AKT and p-p70S6K1 were normalized to levels of total AKT and total p70S6K1, respectively; and levels of MMP-2 and MMP-9 were normalized to those of β-actin; and the results were presented as mean ± SD (*n* = 10). * indicates significant difference compared to the control (*p* < 0.05); (**g**) Tumor sections were prepared for immunohistochemical staining using antibodies against p-AKT and MMP-9. Representative images from the control group and high dose apigenin-treated group are shown (200×); (**h**) OVCAR-3 cells were cultured in RPMI 1640 medium. The expression levels of MMP-2 and MMP-9 were measured by immunoblotting in the cells treated by the solvent and different doses of apigenin for 48 h; (**i**) OVCAR-3 cells were infected by adenovirus carrying Myr-AKT (Ad-Myr-AKT) or GFP vector (Ad-GFP) at 50 MOI for 48 h, followed by the treatment with apigenin at different doses for 48 h. The protein levels of total AKT, MMP-2, and MMP-9 were detected by immunoblotting.

The AKT/p70S6K1 signaling pathway plays an important role in hepatocyte growth factor (HGF)-induced cell invasion and metastasis [15]. Our previous study also demonstrated the important role of PI3K/AKT/mTOR/p70S6K1 pathway in cell migration and invasion in OVCAR-3 cells [10]. To determine what signaling molecules and pathways were affected in tumors from mice treated with solvent or apigenin, we found that apigenin treatment greatly decreased levels of AKT phosphorylation, but did not affect total AKT, total and p-p44/42-MAPK levels (Figure 5a,b). Apigenin treatment also decreased the expression levels of p-p70S6K1, but not total p70S6K1 expression (Figure 5c,d). Matrix metalleproteinases (MMPs) are a family of endopeptidases that are associated with tumor cell invasion and metastasis [16]. Their major contribution to cancer development is to degrade ECM molecules for promoting cancer cell migration/invasion across tissues boundaries [17]. Increased expression of both MMP-2 and MMP-9 in human ovarian cancer cells is associated with metastasis potential and poor prognosis of the disease [15]. By contrast, the down-regulation of both proteins by antisense strategies or by overexpression of natural inhibitors inhibits metastasis [18]. In this study, we found that the expression levels of MMP-9, but not MMP-2, in ovarian tumor tissues were significantly decreased in apigenin-treated groups (Figure 5e,f). Similar results were obtained by immunohistochemistry analysis that showed that apigenin treatment greatly diminished the expression levels of MMP-9 and p-AKT in the tumor tissues (Figure 5g). It was reported that MMP-9 can be activated by fibronectin via PI3K-AKT pathways in ovarian cancer cells [19]. To further determine whether the inhibition of MMP-9 by apigenin was mediated by AKT, we infected OVCAR-3 cells with adenovirus carrying GFP or Myr-AKT, an active form of AKT. As shown in Figure 5i, total AKT was overexpressed in cells after infection. However, the inhibitory effect of apigenin on MMP-9 was rescued in the cells constitutively expressing Myr-AKT. This indicated that AKT is an essential upstream molecule of MMP-9 expression which is inhibited by apigenin.

## 3. Materials and Methods

### 3.1. Chemicals and Antibodies

Apigenin was purchased from Shanghai Winherb Medical Science Co (Shanghai, China). The antibodies against phospho-AKT (Ser473), total AKT, p70S6K1, β-actin and phospho-p44/42MAPK (Thr202/Tyr204) were purchased from Cell Signaling Technology (Beverly, MA, USA). The antibodies against MMP-2(L638) and MMP-9(W680) were purchased from Santa Cruz Biotechnology (Santa Cruz, CA, USA).

### 3.2. Animal Model and Treatment

Six-week-old female nude mice (BALB/cA-nu (nu/nu)) were purchased from Shanghai Experimental Animal Center (Chinese Academy of Sciences, China), and maintained under pathogen-free conditions. All procedures involving animals were approved by the Institutional Committee on Animal care, Nanjing Medical University. The mean weight of animals on arrival was 20 ± 2 g (mean ± SD). OVCAR-3 cells were cultured to 90% confluence, trypsinized, washed twice with 1× PBS, and resuspended in serum-free medium. Mice were placed under general anesthesia, ovaries were exposed and 20 μL of resuspended cells (1 × 10^6^ cells) were injected onto the ovarian capsule via sterile microsyringe.

All the mice were divided into three groups, one control and two treatment groups (*n* = 10). The apigenin solved in 0.2% gelatin were given daily by intragastric administration at doses of 75 mg/kg and 150 mg/kg. The mice that received the same volume of 0.2% gelatin served as the control group. The apigenin treatments started one week prior to implantation and continued until the mice were sacrificed. Tumor growth (abdominal distention) and mouse weight were monitored twice a week. Four weeks post-implantation, the mice were sacrificed by decapitation. Tumors and organs were removed and sectioned in two parts. One part was fixed in Bouin’s fixative solution (saturated picric acid 300 mL, formaldehyde 100 mL, glacial acetic acid 20 mL), and embedded by paraffin. The other part was snap frozen in liquid nitrogen and stored at −80 °C for analysis.

### 3.3. Micrometastasis Assay Based on Genomic PCR for Alu Sequence

In brief, genomic DNA of tissues were extracted by phenol/chloroform and precipitated by ethanol, dissolved in TE buffer and stored at 4 °C. Specific primers for human Alu sequences were Alu-sense 5′-CGAGGCGGGTGGATCATGAGGT-3′ (positions 48–69) and Alu-antisense 5′-TCTGTCGCCCAGGCCGGACT-3′ (positions 273–254) [20]. PCR was carried out using 100 ng of genomic DNA as template and recombinant Taq DNA polymerase with the following thermocycling conditions: an initial denaturation step of 94 °C for 4 min; 25 cycles comprising 94 °C for 30 s, 60 °C for 45 s, and 72 °C for 45 s; and a final extension step of 72 °C for 7 min.

### 3.4. Immunoblotting

Harvested cells were washed in cold 1× PBS buffer, and lysed in ice-cold radioimmunoprecipitation assay (RIPA) buffer supplemented with protease inhibitors on ice for 30 min. Cell debris was discarded by centrifugation at 12,000 rpm for 10 min at 4 °C. The protein concentrations were assayed using Bio-Rad protein assay reagents. Protein samples were subjected to immunoblotting analysis.

### 3.5. Immunohistochemistry Analysis

Tumor tissues were sliced and fixed in Bouin’s fixative solution for 24 h, and processed by the paraffin-embedded method. The paraffin-embedded tumor tissues (5 μm thick) were heat-immobilized and deparaffinized using xylene, then rehydrated in a series of increasing ethanol concentrations. In order to reduce background staining, the Dako Envision two-step method of immunohistochemistry was used. In brief, the sections were washed three times in 1× PBS buffer, and incubated for 10 min in 0.3% hydrogen peroxide. Antigen retrieval was performed by boiling the sections in 10 mM citrate buffer (pH 6.0) by microwave for 15 min, followed by incubation with primary antibodies: mouse anti-human CA125, rabbit anti-human p-AKT (1:100) and MMP-9 (1:200) at 4 °C overnight. After three washes, the sections were incubated with HRP-labeled goat anti-rabbit antibody (DAKO; Hamburg, Germany) for 30 min. The tumor tissue sections were incubated with 3,3′-diaminobenzidine (DAB) for 5~10 min, and detected under a light microscope. Sections incubated with PBS buffer instead of the primary antibodies were used as a negative control.

### 3.6. Cell Culture

Human ovarian cancer cells OVCAR-3 (American Type Culture Collection, Manassas, VA, USA) were cultured in RPMI 1640 medium containing 10% heat-inactivated fetal bovine serum (HI-FBS; Gibco BRL, Grant Island, NY, USA), 100 units/mL penicillin G, and 100 μg/mL streptomycin.

### 3.7. Wound-Healing Assays

OVCAR-3 cells were cultured to 100% confluence in 6-well plates. The wound-healing assays were performed using a sterile 200 μL pipette tip to scratch the cells to form a wound. Cells were washed once with cold 1× PBS buffer, and treated with 10 μM apigenin or solvent DMSO alone for 48 h. Migration of the cells was evaluated at 0 and 48 h with an inverted Olympus phase-contrast microscope.

### 3.8. Matrigel Transwell Invasion Assay

OVCAR-3 cells were cultured to near confluence in RPMI 1640 medium containing 10% FBS. The cells were harvested and washed in RPMI medium without serum. The cells were suspended in RPMI 1640 medium at 5 × 10^5^ cells/mL. Each Transwell of 24-well plate was pre-coated with 50 μL Matrigel. RPMI 1640 medium (600 μL) containing 10% FBS was added to each well (lower compartment), and 0.1 mL (0.5 × 10^5^ cells) of cell suspension was added onto each Transwell insert (upper compartment). The plates were incubated for 48 h at 37 °C. The invaded cells on the bottom surface of the membrane were fixed by dehydrated alcohol, and stained by 0.2% crystal violet solution (Sigma-Aldrich, USA). After the wash, cells were photographed at 400× magnification. Then the stained cells were eluted by 20% glacial acetic acid for 20 min. The elutes were measured with a microplate reader (Bio-Dad, USA) at 570 nm.

### 3.9. Statistical Analysis

The results represent mean ± SD from three independent experiments. Student’s *t* test, One-way ANOVA and χ^2^*-*test were used for statistical analysis by SPSS 11.5 for Windows. Differences were considered significant at a value of *p* < 0.05.

## 4. Conclusions

The anti-tumor potential of the naturally occurring flavonoid apigenin has been extensively studied in several cancer cells. However, the prevention and therapeutic effects of apigenin through oral administration are unknown. We developed an orthotopic ovarian tumor model and found that apigenin oral administration inhibited ovarian tumor micrometastasis in liver, lung, small intestine and stomach in different degrees. We also found that apigenin specifically inhibited MMP-9, but not MMP-2 expression in both tumor tissues and cancer cells. We found that AKT is an essential molecule for apigenin-inhibited MMP-9 expression. AKT may transmit the signal through p70S6K1 to activate MMP-9 expression. Since most patients (80%) with ovarian cancer have metastatic disease [21], these findings reveal a protective role of apigenin in cancer metastasis via oral uptake, highlighting a new rationale for apigenin in ovarian cancer prevention and treatment in the future.

## Figures and Tables

**Table 1:** The numbers of micrometastases in four different organs from three groups. The total numbers of mice with detectable human ovarian cancer cells in specific organs from three groups are shown with each group containing 10 mice.

Group	Lung	Small intestine	Liver	Stomach
Control	4/10	7/10	7/10	6/10
Low dose (75 mg/kg)	3/10	5/10	6/10	2/10 *

High dose (150 mg/kg)	3/10	3/10 *	5/10	3/10 *

* indicates significant difference (*p* < 0.05, χ^2^-test).
